# Comparable Long-Term Rabies Immunity in Foxes after IntraMuscular and Oral Application Using a Third-Generation Oral Rabies Virus Vaccine

**DOI:** 10.3390/vaccines9010049

**Published:** 2021-01-14

**Authors:** Verena te Kamp, Virginia Friedrichs, Conrad M. Freuling, Ad Vos, Madlin Potratz, Antonia Klein, Luca M. Zaeck, Elisa Eggerbauer, Peter Schuster, Christian Kaiser, Steffen Ortmann, Antje Kretzschmar, Katharina Bobe, Michael R. Knittler, Anca Dorhoi, Stefan Finke, Thomas Müller

**Affiliations:** 1 Institute of Molecular Virology and Cell Biology, Friedrich-Loeffler-Institut (FLI), WHO Collaborating Centre for Rabies Surveillance and Research, OIE Reference Laboratory for Rabies, 17493 Greifswald-Insel Riems, Germany; verena.te_kamp@boehringer-ingelheim.com (V.t.K.); conrad.freuling@fli.de (C.M.F.); madlin.potratz@fli.de (M.P.); antonia.klein@fli.de (A.K.); Luca.zaeck@fli.de (L.M.Z.); Elisa.Eggerbauer@gmx.de (E.E.); stefan.finke@fli.de (S.F.); 2 Boehringer Ingelheim GmbH, 55216 Ingelheim am Rhein, Germany; 3 Institute of Immunology, Friedrich-Loeffler-Institut (FLI), 17493 Greifswald-Insel Riems, Germany; virginia.friedrichs@fli.de (V.F.); michael.knittler@fli.de (M.R.K.); anca.dorhoi@fli.de (A.D.); 4 Ceva Innovation Center, 06861 Dessau-Rosslau, Germany; ad.vos@ceva.com (A.V.); peter.schuster@ceva.com (P.S.); christian.kaiser@ceva.com (C.K.); steffen.ortmann@ceva.com (S.O.); antje.kretzschmar@ceva.com (A.K.); katharina.bobe@ceva.com (K.B.); 5 Thüringer Landesamt für Verbraucherschutz, 99947 Bad Langensalza, Germany

**Keywords:** foxes, oral vaccination, SPBN GASGAS, neutralizing and binding antibodies, immunoglobulin isotypes, interferon gamma

## Abstract

The live genetically-engineered oral rabies virus (RABV) variant SPBN GASGAS induces long-lasting immunity in foxes and protection against challenge with an otherwise lethal dose of RABV field strains both after experimental oral and parenteral routes of administration. Induction of RABV-specific binding antibodies and immunoglobulin isotypes (IgM, total IgG, IgG1, IgG2) were comparable in orally and parenterally vaccinated foxes. Differences were only observed in the induction of virus-neutralizing (VNA) titers, which were significantly higher in the parenterally vaccinated group. The dynamics of rabies-specific antibodies pre- and post-challenge (365 days post vaccination) suggest the predominance of type-1 immunity protection of SPBN GASGAS. Independent of the route of administration, in the absence of IgG1 the immune response to SPBN GAGAS was mainly IgG2 driven. Interestingly, vaccination with SPBN GASGAS does not cause significant differences in inducible IFN-γ production in vaccinated animals, indicating a relatively weak cellular immune response during challenge. Notably, the parenteral application of SPBN GASGAS did not induce any adverse side effects in foxes, thus supporting safety studies of this oral rabies vaccine in various species.

## 1. Introduction

The widespread use of inactivated rabies vaccines has dramatically reduced rabies in domestic animals and humans worldwide [1]. Inactivated rabies virus (RABV) vaccines are deemed highly effective in many species including wildlife [2,3,4]. However, for reasons of practical application, they are no alternative for mass vaccination of wildlife rabies reservoirs [5]. Oral rabies vaccines comprising attenuated and modern biotechnology-derived vaccine constructs have leveraged new strategies for wildlife rabies control (reviewed in [5]). Live-attenuated RABVs are known to replicate rapidly, express large amounts of glycoprotein (G), and induce strong immune responses that can clear a RABV infection [6,7,8,9]. Their efficacy has been demonstrated in a variety of wild carnivore reservoir species [10,11,12,13,14,15,16,17,18,19,20,21,22,23]. Conventionally, the effectiveness of rabies vaccines in wildlife target species is determined by serological testing [24], e.g., the detection of rabies virus neutralizing (VNA) or binding antibodies as a surrogate for humoral immune response and hence, adequate B-cell response [25]. Apart from that, independent of the humoral response, survival after the challenge is another criteria for licensing [26,27]. Recently, the analysis of canine T- and B-cell response to RABV antigen was suggested as an alternative to in vivo efficacy studies for inactivated rabies vaccines for dogs [28,29]. This underscores the fact that the immune system with its cellular and humoral mechanisms as well as genetic imprinting is complex and needs more thorough consideration if full immune response to oral rabies vaccines and protection from a lethal challenge is assessed [30].

Next to the adaptive immune response, there is evidence that attenuated RABVs also activate the host innate immune responses [31]. Notably, virus gene expression in lymphoid tissue cells after oral vaccine uptake [32] and the ensuing cytokine induction, antigen processing, and presentation are crucial for the recruitment of antigen-presenting cells (APC), in particular dendritic cells (DCs), for initiation and shaping of adaptive T- and B-cell mediated immune responses [33]. Initiation of CD4+ and CD8+ T cell responses after RABV infection independent of the virus used has been described [34], with live RABV vector vaccines able to elicit both a type 1 and 2 immune response [35]. Hence, immunization with live-attenuated vaccines is supposed to provide long-lasting rabies immunity, superior to the protection induced by inactivated vaccines [36]. Most of the studies to evaluate the innate host immune responses to live-attenuated RABVs, including oral rabies vaccines, were carried out in small laboratory animals, not the natural hosts for the rabies virus. Only Gnanadurai et al. studied innate and humoral immune response in dogs after infection with a third generation attenuated rabies vaccine virus in more detail [37].

In contrast, despite foxes’ relevance as an important primary reservoir host for rabies in the Northern Hemisphere, immunity to RABV remains scantly explored in this carnivore species. This is due to (i) the initial success of oral rabies vaccines in protecting foxes from infection, (ii) the impracticability of foxes as an animal model and (iii) the paucity of reliable immunological tools. In fact, rabies-specific immune parameters were only partially examined. The few studies available mainly focused on the induction of IgM and IgG class antibodies after intramuscular, intestinal, and oral administration of inactivated rabies vaccines [38,39,40]. In contrast, T-cell response was investigated only after oral vaccination using modified and recombinant life virus vaccines, e.g., SAG2 and VRG [41]. No direct comparative evaluation of those immune parameters after parenteral and oral application of a modified life virus rabies vaccine in foxes had been conducted yet. Species-specific differences in IgG subclasses may have different effector functions and a unique profile with respect to antigen binding, immune complex formation and triggering of effector cells. Further considering that an IgG antibody response to different types of antigens leads to marked skewing toward particular subclasses [42], studying IgG subclasses will help to better understand the underlying mechanisms of immune response in particular reservoir species. Therefore, in the present study, we investigated immune responses in red foxes (*Vulpes vulpes*) after oral and parenteral vaccination with the third generation vaccine strain SPBN GASGAS with a particular focus on the kinetics of rabies-specific antibodies, including antibody isotype/classes and subclasses as well as on antigen-specific T-cell activity prior and after challenge.

## 2. Materials and Methods

### 2.1. Study Design and Sampling

The experimental setup was designed as a blinded study following Good Clinical Practice (GCP) guidelines [43]. A total of twenty-two female captive-reared, seronegative foxes aged 2–3 months were randomly allocated to three treatment groups. Animals in group 1 (*n* = 11) were part of a long-term efficacy study and received a vaccine bait containing the vaccine strain SPBN GASGAS (Genbank accession number MH660455 titer of 10^6.6^ FFU/mL) [20], while animals in group 2 (*n* = 6) were inoculated parenterally with 0.5 mL of the vaccine with the same titer in the gluteus muscles of the right hind leg. Group 3 (*n* = 5) received a placebo-bait. Information on the source of animals, housing, feeding, general care, basic vaccination (canine distemper), challenge, anesthesia, and euthanasia of the animals is provided in detail elsewhere [19]. Briefly, blood samples were taken prior (day 0) and after vaccination at different time points, i.e., 14, 28, 58, 118, 178, 268, 365, 379, 393 and 455 days post vaccination (dpv) or at the time point of euthanasia with an additional heparinized blood sample taken at 268, 365, 379, 393, 455 dpv for isolation of peripheral blood mononuclear cells (PBMCs).

At 365 dpv, all animals were challenged intramuscularly (*Musculus masseter*) with 10^3.0^ MICLD50/dose of rabies field virus strain “fox Krefeld” (FLI ID 148¸ Genbank accession number LN879481.2) [19,20] and monitored for 90 days post infection (dpi). Humane clinical endpoints were defined as described elsewhere [44]. All surviving animals were euthanized 90 dpi (equals 455 dpv) as described [19,20]. The study was conducted in accordance with national and European regulations, and European guidelines on animal welfare from the Federation of European Laboratory Animal Science Associations (FELASA) [20]. The animal study was approved by the Ethics Committee of the Federal State of Mecklenburg-Western Pomerania, Landesamt für Landwirtschaft, Lebensmittelsicherheit und Fischerei Mecklenburg-Vorpommern, 18003 Rostock, Germany (FLI-7221.3-1-087/16).

### 2.2. Antigen Detection and Serological Assays

After challenge, brain samples from all diseased animals and survivors were tested for the presence of RABV antigen and viral RNA using the direct fluorescent antibody test (FAT) [45] and RT-qPCR [46], respectively. Rabies-specific VNA were detected using the rapid fluorescent focus inhibition test (RFFIT) and converted into international units (IU) per mL as described elsewhere [25]. VNA titers ≥ 0.5 IU/mL were considered seropositive. Binding antibodies were detected by a commercial blocking ELISA (BioPro Rabies ELISA, Prague, Czech Republic) according to manufacturer specifications with a percentage blocking (PB) value of ≥40% used as a threshold for positivity [47].

### 2.3. Serum Antibody Isotyping

The antibody classes and subclasses were determined by a cell-based ELISA using SPBN GASGAS as antigen and dog-specific, horseradish-peroxidase (HRP) labeled total anti-IgA, anti-IgM, anti-IgG1, and anti-IgG2 (Bethyl Laboratories INC, Montgomery, AL, USA) secondary antibodies, as described previously [48,49]. ELISA results were presented as fold increase with values obtained at 0 dpv used for normalization. The reactivity of the antibodies beyond the dog was established before in a related canid species, raccoon dog [49], and also assessed in silico by a comparative alignment of fox and dog Ig-related mRNA and protein sequences. Additionally, the specificity of the cell-based in-house ELISA was proven by a commercial dog-specific rabies virus IgM Antibody ELISA Kit (Abcam, Cambridge, UK) using sera of 6 parenterally vaccinated animals selected at different time points, e.g., 0, 14, 28, 58, 365, 379, 393 and 455 dpv.

### 2.4. Isolation of PBMCs

Fox PBMCs were isolated on Ficoll-Paque Plus density gradient as described [41,50]. All heparinized blood was briefly diluted with phosphate-buffered saline containing 2% fetal bovine serum (PBS/2% FBS) in equal quantities. For each blood sample (5–9 mL), 15 mL Ficoll^®^ Paque Plus density gradient medium (GE Healthcare, Waukesha, WI, USA) was filled into a SepMate™-50 centrifugation tube (StemCell Technologies, Inc., Vancouver, BC, Canada) before adding the diluted blood sample on top of the gradient. After centrifugation for 10 min at 1200× *g*, the PBMC containing fraction was poured off into a clean 50 mL tube and washed 3 times with PBS/2% FBS for 8 min at 300× *g*. Isolated PBMCs were then suspended in RPMI-1640 medium (Gibco™ [Thermo Fisher Scientific], Waltham, MA, USA) supplemented with FBS (10%), GlutaMAX™-I (2 mM; Gibco™), non-essential amino acids (1%; Gibco™), Penicillin/Streptomycin (100 IU/mL/100 µg/mL; Gibco™), Sodium Pyruvate (1 mM; Gibco™), Gentamicin (20 µg/mL; Gibco™) and 2-Mercaptoethanol (50 µM; Gibco™).

### 2.5. IFN-γ Enzyme-Linked ImmunoSpot (ELISpot) Detection Assay

To measure antigen specific T-cell activity before and after challenge, Interferon-gamma (IFN-γ) secretion was analyzed using the Canine IFN-γ ELISpotPlus (ALP) Kit (Mabtech AB, Nacka Strand, Sweden). Briefly, PBMCs extracted from whole blood were stimulated with two concentrations (2.5 and 5.0 µg/mL) of the inactivated (0.05% ß-propiolactone, sonicated) RABV strain SAD L16 [51] and subsequently transferred in duplicate (2 × 10^5^ PBMCs/cavity) to pre-coated, equilibrated 96-well ELISpot plates. Concanavalin A (Con A—3 µg/mL; Sigma-Aldrich-Merck, Darmstadt, Germany) and cell culture medium served as positive and negative control, respectively, in every run. After 24 h of stimulation, secretion of IFN-γ was detected with a biotinylated anti-canine IFN-γ monoclonal antibody, streptavidin-ALP, and ready-to-use BCIP/NBT-plus substrate followed by fixation with 4% formaldehyde for 20 min. Spots were automatically identified using the vSpot Spectrum ELISpot Reader (AID GmbH, Strassberg, Germany) and counted as Spot Forming Units per one million cells (SFU/10^6^ cells). 

### 2.6. Statistical Analysis

Differences in mean percent blocking (MPB) values and geometric mean titers (GMT) of binding antibodies and VNAs, respectively, between groups 1 and 2 at different sampling time points were tested for significance using unpaired T-tests with a significance level of α = 0.05. Statistically significant differences in kinetics of antibody classes and isotypes between consecutive sampling time points within (*) and between treatment groups (#) were analyzed using an ordinary two-way ANOVA test. Differences with *p* < 0.05 were defined as significant (*), *p* < 0.01 as highly significant (**) and *p* < 0.001 (***) plus *p* < 0.0001 (****) as extremely significant. For IFN-γ ELISpot, the calculated SFU/10^6^ cells were normalized to the respective unstimulated controls. Analyses of significance in the differences between two medians were calculated by an ordinary two-way ANOVA followed by Šidák’s multiple comparison test. Differences between two means with *p* < 0.01 were considered highly significant (**) and *p* < 0.001 extremely significant (***). To determine the significance of correlations between (i) numbers of SFU/*10^6^ cells obtained for individual animals and corresponding RFFIT and ELISA results and (ii) VNAs, percentage blocking, and IgG/IgG2, the Pearson correlation coefficient testing was used. Differences with *p* < 0.05 were defined as significant (*), *p* < 0.01 as highly significant (**) and *p* < 0.001 (***) plus *p* < 0.0001 (****) as extremely significant. True outliers were identified by ROUT-test and excluded before calculation, as were seronegative animals within the oral group. All statistical analyses were carried out using Graphpad Prism 7 (GraphPad Software Inc., San Diego, CA, USA).

## 3. Results

### 3.1. Rabies-Specific Antibody Response to Rabies Vaccination

**Figure 1:**
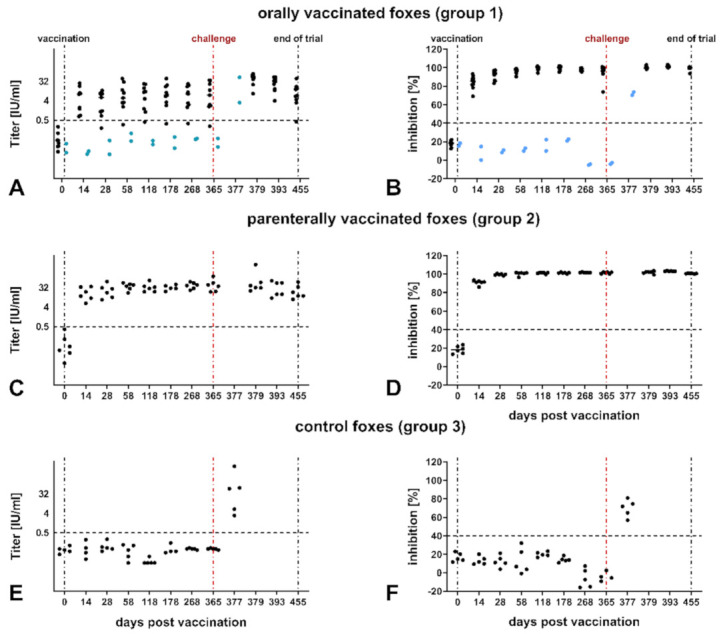
Kinetics of RABV-specific VNA (**A**,**C**,**E**) and binding antibodies (PB, (**B**,**D**,**F**)) after oral and parenteral vaccination with SPBN GASGAS (10^6.6^ FFU/mL) and challenge; (**A**,**B**): orally vaccinated foxes; (**C**,**D**): parenterally vaccinated foxes (group 2); (**E**,**F**): non-vaccinated controls. Serological data of animals that succumbed to RABV infection and had to be euthanized 12–14 dpi (Groups 1, 3) were compiled at 377 dpv. Seronegative foxes (No° 34 and 41) in the orally vaccinated group are shown in blue. Vertical dotted lines indicate time point of vaccination (0 dpv), challenge (365 dpv), and termination of the experiment (455 dpv). Horizontal dotted lines show the positivity threshold for RFFIT (0.5 IU/mL) and ELISA (40% blocking).

Vaccination with SPBN GASGAS induced a strong rabies-specific primary immune response. Fourteen dpv, all orally (Group 1) and parenterally (Group 2) vaccinated foxes developed rabies virus-specific antibodies except for two foxes in the orally vaccinated group (Figure 1, Supplementary Tables S1 and S2). The MPB values of binding antibodies as determined by ELISA showed almost no variation and did not differ between animals of Group 1 and 2 at all sampling time points (Figure 1B,D). In contrast, the VNAs of parenterally vaccinated animals pre-challenge were more consistent and significantly higher (3–4 times; *p* < 0.001) compared to animals from the orally vaccinated group. This difference changed after challenge when the GMTs of animals from the latter group increased to comparable levels (Figure 1A,C, Table S1). Notably, two foxes (No° 34 and 41) of the oral group, as well as the control foxes (Group 3), did not develop a rabies-specific antibodies pre-challenge (Figure 1A,B,E,F).

### 3.2. Survival after Challenge

At the time point of challenge (365 dpv), all parenterally vaccinated foxes were both RFFIT and ELISA positive for rabies VNA and binding antibodies respectively (Figure 1C,D). In comparison, only 9 and 8 of 11 orally vaccinated foxes exhibited RABV-specific binding antibodies and VNAs, respectively, at this time point (Figure 1A,B, Supplementary Tables S1 and S2). All seropositive foxes survived the challenge infection, whereas the control animals and the two seronegative foxes from the oral group (Group 1, No° 34 and 41; Supplementary Tables S1 and S2) succumbed to rabies within 14 days and had to be euthanized (377 dpv). Subsequently, these animals were positive in FAT but notably, demonstrated rabies-specific antibodies at the day of necropsy (Figure 1A,B,E,F). 

### 3.3. Kinetics of RABV-Specific Antibody Classes and Subclasses

Alignment of fox- (*Vulpes vulpes*, VulVul2.0) and dog-(*Canis lupus familiaris*, CanFam3.1) Ig-related mRNA and protein sequences showed an amino acid sequences match of at least 75% (Supplementary Table S3) indicating putative cross-reactivity of commercial anti-dog antibodies with fox immunoglobulins. Specificity and functionality of the cell-based in-house ELISA were confirmed by comparing IgM-specific antibodies of six parenterally vaccinated animals obtained at different dpv in a commercial dog-specific rabies virus IgM ELISA kit. Although the in-house ELISA showed higher optical density (OD) values, similar trends were detected in both assays (Supplementary Figure S1).

**Figure 2:**
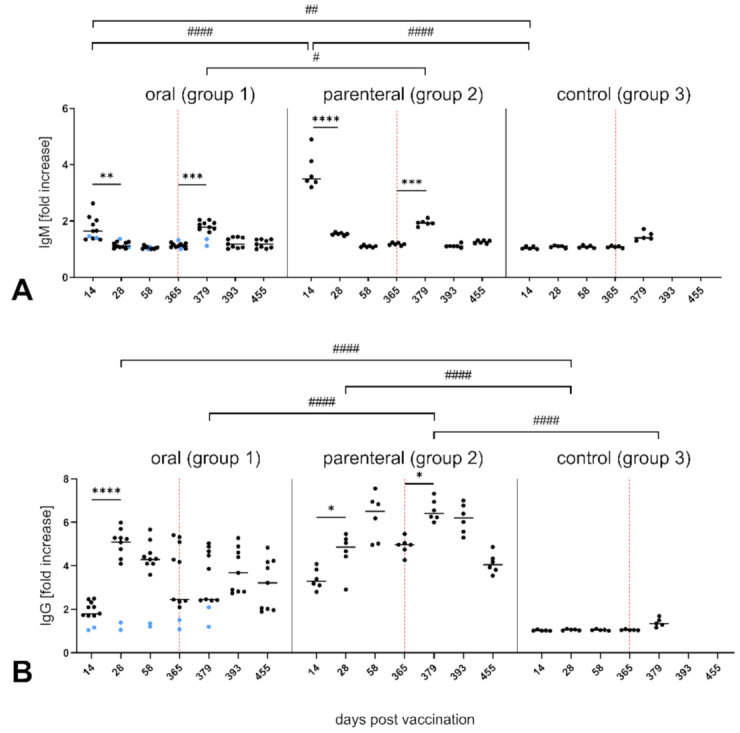
Kinetics of RABV-specific IgM (**A**) and IgG (**B**) in serum of vaccinated and naïve foxes post vaccination and post challenge. Data are expressed as fold change values for individual animals (dots) and as mean (horizontal line) for each sampling time point and group. Seronegative foxes (No° 34 and 41) in the orally vaccinated group are shown in blue. Statistically significant differences between consecutive sampling time points within groups are denoted as follows: * *p* ≤0.01; ** *p* ≤ 0.005; *** *p* ≤ 0.001; **** *p* ≤ 0.0001, while those between groups were denoted by ^#^ *p* ≤ 0.01; ^##^ *p* ≤ 0.005; ^####^ *p* ≤ 0.0001. The vertical dotted line indicates the Time Point of challenge (365 dpv).

RABV-specific IgM antibodies were first detected at 14 dpv irrespective of the route of administration. Parenterally vaccinated animals showed significantly higher IgM levels than the orally vaccinated group. IgM concentrations significantly declined after 4 weeks (28 dpv) in all vaccinated animals and dropped to naïve levels at 58 dpv (Figure 2A). 

Fourteen days post challenge (379 dpv), levels of IgM antibodies significantly peaked again in the vaccinated groups with comparable fold change values. Although the control group showed slightly increased IgM antibodies 12 dpi (377 dpv), they were not significantly different from baseline levels obtained before the challenge. Similarly, no IgM antibodies in the two seronegative foxes (No° 34 and 41) of the oral group that succumbed to infection (379 dpv) could be detected (Figure 2A). 

Elevated levels of total IgG antibodies were first detected 14 dpv with peaks at 28 and 58 dpv in the orally and parenterally vaccinated group, respectively. In general, parenterally vaccinated foxes seemed to develop gradual, yet higher and stable IgG antibody abundancies (Figure 2B). After the challenge, IgG antibodies significantly increased only in parenterally vaccinated animals, while levels of IgG antibodies in orally vaccinated foxes remained mostly unaffected. Control animals and the two seronegative animals from the oral group did not develop IgG RABV-specific antibodies at any time point during the observation period. However, a low-level IgG antibodies after challenge in the control group were noted (Figure 2B). 

**Figure 3:**
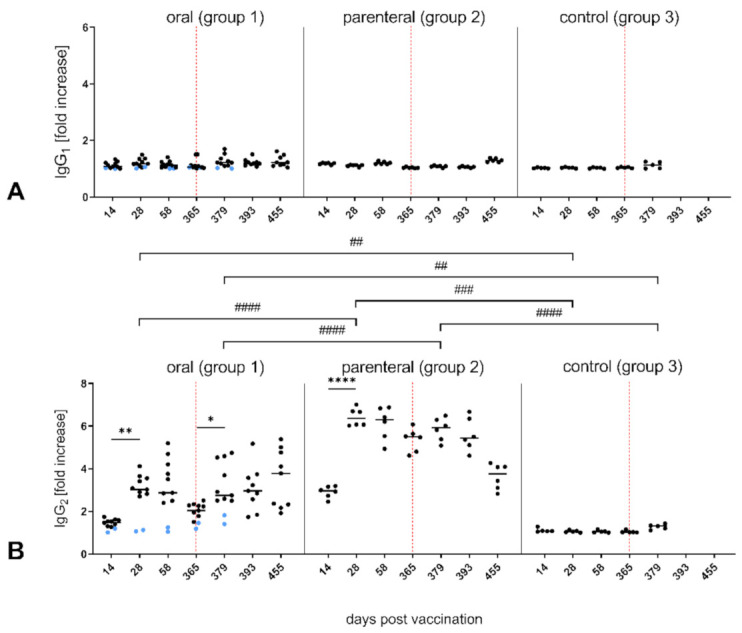
Kinetics of RABV-specific IgG subclasses IgG1 (**A**) and IgG2 (**B**) in serum of vaccinated and naïve foxespost vaccination and post challenge. Data are expressed as fold change values for individual animals (dots) and as mean (horizontal line) for each sampling time point and group. Seronegative foxes (No° 34 and 41) in the orally vaccinated group are shown in blue. Statistically significant differences between consecutive sampling time points within groups are denoted as follows: * *p* ≤0.01; ** *p* ≤ 0.005; **** *p* ≤ 0.0001, while those between groups were denoted by ^##^ *p* ≤0.01; ^###^ *p* ≤ 0.005; ^####^ *p* ≤ 0.001. The vertical dotted line indicates the Time Point of challenge (365 dpv).

When RABV-specific IgG subclasses were investigated, IgG1 antibodies could not be detected in any three treatment groups (Figure 3A). In contrast, foxes in the vaccinated groups (Groups 1, 2) exhibited pronounced IgG2 antibody kinetics similar to those obtained for total IgG RABV-specific antibodies (Figure 2B and Figure 3B), with parenterally vaccinated foxes showing significantly higher IgG2 levels as compared to orally vaccinated ones. 

Strikingly, after challenging, a significant and sustained increase in IgG2 antibodies was exclusively detected in the orally vaccinated group. In contrast, IgG2 antibodies in parenteral vaccinated foxes dropped to comparable levels only at the end of the study (455 dpv). Again, the two seronegative foxes from the oral group showed no IgG2 RABV-specific antibodies, as did the control group (Figure 3B).

**Figure 4:**
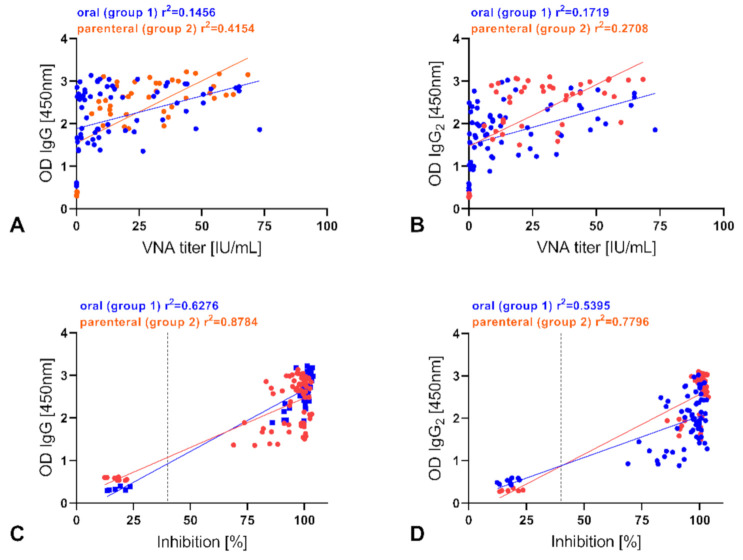
Correlation between RABV-specific VNAs (RFFIT), binding antibodies (ELISA), and RABV-specific IgG and IgG2 in orally and parenterally vaccinated foxes. Pearson correlation coefficients were calculated for VNA titers and IgG (**A**) and IgG2 (**B**) levels as well as between PB and IgG (**C**) and IgG2 (**D**) levels. Dots represent values from an individual animal for different time points (listed in method section). ROUT-test identified real outliers. The vertical dotted line indicates the threshold of positivity in ELISA (40%).

Comparing IgG RABV-specific antibodies with VNA titers and PB values of binding antibodies from vaccinated animals revealed significant positive correlations (*p* ≤ 0.0001) between IgG, IgG2 antibodies, and both VNA (Figure 4A,B) and binding antibodies (Figure 4C,D and Figure S2). Along with these findings, correlations calculated for control animals were all significant as well.

### 3.4. Rabies-Specific IFN-γ Release after Rabies Vaccination and Challenge Infection

**Figure 5:**
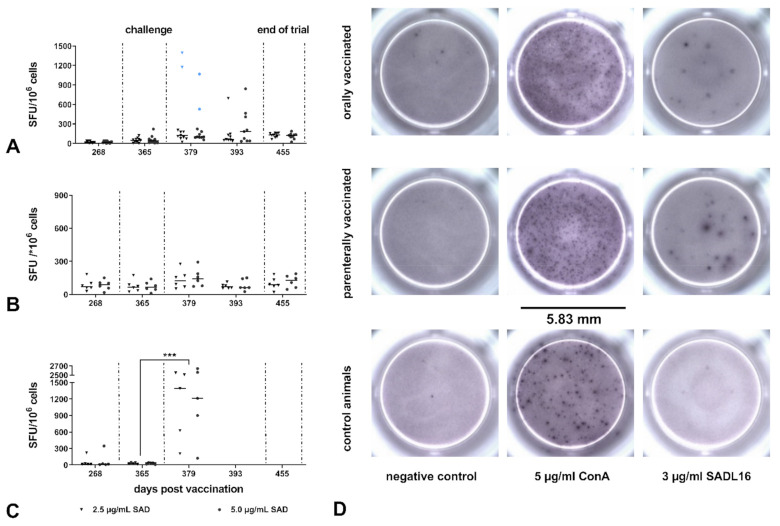
T-cell specific IFN-γ production of PBMCs from foxes after rabies vaccination and challenge infection: (**A**) orally vaccinated foxes; (**B**): parenterally vaccinated foxes; (**C**) non-vaccinated controls. Data are depicted as IFN-γ SFU of PBMCs from individual foxes in response to stimulation with 2.5 µg/mL (triangles) and 5.0 µg/mL (dots) of RABV antigen (SAD L16) and mean (horizontal line) for each time point and group. Seronegative foxes (No° 34 and 41) in the orally vaccinated group are shown in blue. Statistically significant differences between consecutive sampling time points within groups are denoted as follows: *** *p* ≤ 0.001. The vertical dotted lines indicate the time point of challenge (365 dpv) and the study’s termination (455 dpv). (**D**) Representative images of cavities showing IFN-γ SFU of ConA and RABV antigen-stimulated fox PBMCs for vaccinated and control groups.

Despite individual variability in responses, foxes of both vaccinated groups showed a relatively low T-cell specific immune response before challenge, indistinguishable from the control group (Figure 5A–C). With a mean of 51 and 80 SFU/*10^6^ cells (both concentrations of 2.5 and 5.0 µg/mL RABV antigen combined), IFN-γ secretion in the orally and parenterally vaccinated group, respectively, did not differ. Comparatively, the stimulation of PBMCs with ConA resulted in a robust IFN-γ secretion of about 790 SFU/*10^6^ cells on average (Supplementary Table S4).

The challenge infection did not significantly affect IFN-γ secretion to RABV in any of the vaccinated fox groups. Although the mean number of SFU/*10^6^ cells in the orally and parenterally vaccinated groups increased to 144 and 110 after the challenge (Table S3), respectively, this difference was not significant when mean values from 379 dpv (14 dpi) and 365 dpv (day of the challenge) were compared within both groups (Figure 5A,B). There was also no difference in IFN-γ SFU between the two vaccinated groups at the respective time points, nor did the values differ from those of the control group pre-challenge. The IFN-γ secretion did not match with the VNA titers or levels of rabies-specific binding antibodies obtained. In contrast, as a result of challenging infection in the control group, the mean number of SFU/*10^6^ cells significantly increased (*p* < 0.0002) from 37 (286, 365 dpv) to 1297 (377 dpv) on average (Figure 5C). 

## 4. Discussion

Consistent with previous reports [12,15,20,21,41], we demonstrate comparable long-term (365 dpv) rabies immunity in foxes after intramuscular and oral application using a third-generation oral rabies virus vaccine, i.e., SPBN GASGAS. The humoral immune response resembles those reported in previous studies with the same and other modified live virus vaccines (Figure 1) [18,19,20,48,52,53,54,55]. With the exception of two animals, there was no difference in binding antibody (Figure 1B,D) and VNA response (Figure 1A,C) between both vaccinated groups. However, as expected and in agreement with previous reports in mice and dogs [6,37], our study shows that intramuscular delivery of SPBN GASGAS in foxes induced higher and more consistent levels of VNA in comparison to oral administration (Figure 1A,C). A decrease in VNA resulting from a shift in production of circulating antibodies to development of memory immune cells [56] as observed in dogs [48,55] could not be observed in our or other previous studies in foxes and raccoon dogs [19,20,41]. The fact that two animals did not mount a measurable immune response after oral vaccination is likely due to inadequate or missing contact with the vaccine itself, although all animals from this group were assumed vaccinated as baits and blisters were not discovered [20]. Alternatively, there is no indication for an underlying immunosuppression in those two seronegative animals, since they responded immunologically similar to control animals after challenge infection.

Using a previously established cell-based ELISA [48], we accurately monitored the kinetics of RABV-specific IgM, IgG, and IgG subclasses IgG1 and IgG2 in foxes and confirmed cross-reactivity of α-dog-immunoglobulin antibodies with fox counterparts [57]. We found commonalities in peaks of VNAs post vaccination. The overall kinetics of IgM and total IgG antibodies in foxes vaccinated with SPBN GASGAS resembles those obtained in dogs vaccinated against canine parvovirus, distemper virus, and RABV [48,58,59,60]. The IgM peak observed in vaccinated animals 14 days after challenge (379 dpv) was unexpected (Figure 2). Given the fact that SPBN GASGAS exhibits 123 (92% identity) and 34 (93% identity) exchanges in the domain coding for G protein at the nucleotide and amino acid level, respectively, compared to the challenge RABV strain fox 148 [61], we speculate that this likely is a response to initial exposure to the slightly differing antigenic features from the challenge strain. 

It is generally assumed that immunization with inactivated RABVs elicits a species independent type-2 response with high levels of type-2-associated IgG1 RABV-specific antibodies [28,29], while live attenuated RABV vaccines induce type-1 responses [36,62]. The results presented here as well as in previous studies support the predominance of type-1 immunity protection of live-attenuated RABVs and modified live vectored RABV vaccines in vaccinated mice [63] and carnivores, including foxes [36,37,48], since independent of the route of administration the immune response to SPBN GASGAS was mainly IgG2 driven (Figure 3). This was also reflected by the significant correlation between total IgG and IgG2 levels, which are critical for protection against lethal rabies infection [64,65] with VNA and binding antibodies (Figure 4). However, the observed differences in IgG and IgG2 antibody patterns (Figure 4) may indicate the presence of additional subclasses, e.g., IgG3, IgG4, within the IgG pool or unspecific signals. While an IgG3 antibody response is considered to be more reflective of a type-1 immunity as detected after vaccination with inactivated rabies vaccines in dogs [29] and humans [66], the role of those two IgG subclasses after oral vaccination is not fully understood. Whether commercially available antisera for detection of canine IgG3 and IgG4 [67] are cross-reactive with foxes immunoglobulins is not clear. 

Considering that wild-type RABV is efficiently protected from clearance by an almost exclusive intraneuronal spread, the capacity to stimulate RABV-specific type-1 immunity mediating antibody-dependent cellular cytotoxicity (ADCC) or complement-dependent cytotoxicity (CDC) is an essential basis for an effective rabies vaccine [33]. Therefore, single immunization with live-attenuated vaccine confers superior, long-lasting protection against wild-type virus challenge compared to inactivated vaccines [36]. It seems that the anti-dog-IgG2 used in our study recognizes an IgG subclass that is thought to induce strong ADCC activity, whereas dog IgG1 does apparently not [63]. Although we cannot formally exclude functional differences between respective IgGs in domestic and wild carnivores, our results provide evidence that protective RABV-specific immunity in foxes might be based on IgG2 leading to ADCC activity and virus neutralization. Follow-up studies will be performed to clarify this interesting point. Although we cannot exclude differences for IgG3 and IgG4 isotypes, our data exclude significant influences of the primary target cells for vaccine virus infection in the mucosa or lymphoid tonsil tissues [32] and muscle tissue. However, further efforts are required to fully understand antibody-related RABV protection, as there seem to be exceptions from the rule. For example, a mixed type-1/type-2 and predominant type-1 response to inactivated RABV have been reported for mice and dogs, respectively [36,48]. Additionally, contradictory results with type-2-based immune responses with high IgG1/IgG2 ratios in mice vaccinated with live recombinant RABV vaccine have been reported [35]. Therefore, interpretation of results is difficult considering that immunogenicity is often investigated using mice as an animal model, which have a different immune system compared to most other species [68].

Interestingly, the perceived advantage in serum VNA induction in foxes parenterally vaccinated with SPBN GASGAS was simultaneously leveled out by the oral group after challenge (Figure 1), indicating that the level of VNAs as an indicator for protection against wild-type RABV infection might have only minimal relevance. There is only a conditional correlation between protection against RABV infection and the level of VNAs [69,70], suggesting the involvement of antibody-dependent functions uncoupled from neutralizing capacity. For any vaccine, T-/NK-cell activation is one of the most consistent properties of anti-rabies immunity [71,72,73]. While CD8+ T-cells are associated with vaccine-induced viral clearance, CD4+ T-cells are necessary to induce rabies-specific antibodies [73]. In particular, alterations in cytokine profiles delivered by T-cells to antigen-stimulated B-cells may also contribute to differences in antibody production following vaccination. For example, PCR analysis showed concomitant expression of interleukin (IL)-4 and IFN-γ in PBMC of foxes vaccinated with SAG2 and VRG [41]. We focused on IFN-γ as an indicator of cellular immunity. It is expected that a vaccine based on attenuated rabies virus will have an activating effect on this arm of the immune defense. However, in contrast to inactivated vaccines in dogs [28,29], vaccination with SPBN-GASGAS did not cause significant differences in IFN-γ release by PBMCs in vaccinated versus non-vaccinated animals, except after challenge (Figure 5). This might indicate a relatively weak cellular immune response during challenge in vaccinated foxes. It is tempting to speculate that, in these seroconverted animals, circulating antibodies as well as a rapidly reacting B-cell memory with preformed RABV-neutralizing IgGs cause efficient rapid virus elimination and thus does not trigger a detectable contribution of IFN-γ-based antiviral defense. In fact, in support of this notion, we observed that the non-vaccinated controls and the two animals with no detectable antibody response (Figure 1) after oral vaccination are the only ones who respond to challenge virus with a measurable IFN-γ release (Figure 5). Clearly, further detailed experiments are necessary to prove such an immunological scenario in vaccinated foxes.

## 5. Conclusions

Although a single intramuscular application of the live recombinant RABV vaccine strain SPBN GASGAS in foxes induces a more robust and sustained production of RABV-VNAs, it is not superior to a single oral application in terms of long-term protection against challenge with an otherwise lethal dose of the wild-type RABV. The seemingly subordinate or short-lived role of T cell-mediated immunity rather suggests a very strong B-cell memory to be responsible for the protective immune response elicited by this particular live recombinant RABV vaccine strain.

## Data Availability

The data presented in this study are available in supplementary material.

## Supplementary Materials

The following are available online at https://www.mdpi.com/2076-393X/9/1/49/s1, Table S1: Individual RABV-specific VNA titers (IU/mL) obtained at different time points post vaccination as measured by RFFIT. Table S2: Detection of individual RABV-specific binding antibodies obtained at different time points post vaccination as measured by ELISA. Table S3. In silico analysis of consensus between Ig-related mRNA and protein sequences of fox and dog. Table S4: Individual numbers of IFN-γ specific SFU/10^6^ cells counted at different time points pre-challenge and post infection after stimulation with 2.5 and 5.0 µg/mL antigen (RABV SAD L16). Figure S1. Detection of RABV-specific IgM in parenterally vaccinated foxes.
