# Supplementary material for: Comparable Long-Term Rabies Immunity in Foxes after IntraMuscular and Oral Application Using a Third-Generation Oral Rabies Virus Vaccine
Source: Vaccines (Basel). 2021 Jan 14;9(1):49. doi: 10.3390/vaccines9010049 (PMC7828770; doi:10.3390/vaccines9010049)
Supplement: Supplementary file 1 [file vaccines-09-00049-s001.pdf]

Supplementary Materials

Table S1. Individual RABV-specific VNA titers (IU/mL) obtained at different time points post vaccination as measured by RFFIT. VNA > 0.5 IU/mL were considered as positive (threshold of positivity).

	**dpv:**	**0**	**14**	**28**	**58**	**118**	**178**	**268**	**365**	**377**	**379**	**393**	**455**
	**Animal ID**												
**Orally vaccinated (Group 1)**	**30**	0.04	19.55	9.36	12.99	10.69	9.81	10.27	35.65	-	40.11	43.4	15.23
	**31**	0.06	8.28	5.83	44.2	24.59	43.83	24.8	36.36	-	63.69	50.66	18.41
	**33**	0.02	8.97	8.48	30.84	24.07	18.57	21.5	24.4	-	64.96	50.66	43.78
	**34**	0.04	0.02	0.06	0.12	0.06	0.08	0.07	0.03	49.8	-	-	-
	**35**	0.03	1.71	1.27	2.13	1.72	2.47	2.69	2.41	-	64.96	32.25	13.72
	**38**	0.13	1.97	0.81	3.41	1.22	3.45	2.03	2.73	-	53.69	14.39	3.28
	**41**	0.02	0.01	0.01	0.05	0.04	0.03	0.06	0.07	3.34	-	-	-
	**42**	0.06	26.56	8.4	9.47	15.83	6.56	8.51	12.85	-	47.72	24.13	7.12
	**43**	0.03	0.76	1.37	3.33	5.02	7.4	3.19	2.2	-	73.14	34.43	6.55
	**44**	0.25	0.84	0.22	0.32	0.42	0.35	0.35	0.27	-	8.76	4.05	0.43
	**45**	0.06	8.28	5.83	44.2	24.59	43.83	24.8	36.36	-	63.69	50.66	18.41
	**GMT**	0.05	1.56	1.14	3.19	2.52	2.90	2.55	2.90	12.90	47.04	27.44	8.38
**Parenterally vaccinated (Group 2)**	**47**	0.38	36.69	19.12	47.55	70.77	20.03	61.53	53.97	-	386.9	68.44	59.74
	**48**	0.13	20.82	11.6	38.29	29.62	29.6	24.79	21.86	-	23.31	54.76	13.55
	**49**	0.01	34.9	31.8	29.33	37.72	46.18	53.32	109.36	-	31.77	53.29	34.9
	**50**	0.06	6.07	62.65	43.92	20.93	21.62	43.83	20.67	-	57.04	10.8	9.35
	**51**	0.04	10.63	28.86	55	34.69	39.68	30.5	43.73	-	40.69	16.04	19.91
	**52**	0.03	13.24	8.93	18.55	21.3	30.89	41.05	38.02	-	33.11	16.15	13.51
	**GMT**	0.06	16.84	22.02	36.57	32.70	29.98	40.58	40.53	-	52.94	28.70	20.35
**Control (Group 3)**	**29**	0.08	0.03	0.07	0.14	0.02	0.06	0.09	0.06	6.32	-	-	-
	**36**	0.05	0.06	0.09	0.02	0.02	0.07	0.08	0.07	57.67	-	-	-
	**37**	0.09	0.24	0.1	0.08	0.02	0.17	0.1	0.14	62.2	-	-	-
	**39**	0.07	0.1	0.25	0.13	0.02	0.07	0.09	0.08	62.75	-	-	-
	**46**	0.13	0.02	0.11	0.04	0.04	0.02	0.09	0.01	3.22	-	-	-
	**GMT**	0.08	0.06	0.11	0.07	0.02	0.06	0.09	0.05	21.49	-	-	-

Table S2. Detection of individual RABV-specific binding antibodies obtained at different time points post vaccination as measured by ELISA. Values are expressed as percent inhibition. RABV PB > 40% inhibition were considered as positive (seroconversion). MPB = Mean percentage blocking values.

	**dpv:**	**0**	**14**	**28**	**58**	**118**	**178**	**268**	**365**	**377**	**379**	**393**	**455**
	**Animal ID**												
**Orally vaccinated (Group 1)**	**30**	18.72	93.23	96.86	97.63	100.68	100.79	98.66	100.14	-	102.09	101.86	99.68
	**31**	22.05	91.21	97.2	98.86	101.61	101.35	99.42	100.78	-	103.01	102.49	100.15
	**33**	18.46	85.65	94.73	98.54	100.17	101.25	99.23	99.37	-	98.36	100.46	100.6
	**34**	15.22	14.7	10.83	9.92	22.15	22.8	-4.31	-4.3	73.63	-	-	-
	**35**	17.92	82.03	86.33	93.97	97.43	96.41	96.79	97.14	-	100.15	100.83	100.68
	**38**	18.19	81.61	85.54	94.83	98.93	97.97	95.41	96.42	-	100.66	100.7	99.51
	**41**	18.46	0.11	8.31	12.92	9.97	20.81	-5.4	-2.83	70.19	-	-	-
	**42**	16.75	78.26	92.19	96.82	98.96	101.76	101.36	98.74	-	99.69	102.29	99.44
	**43**	12.35	84.81	93.68	97.76	100.04	95.07	99.82	73.72	-	99.86	103.31	99.87
	**44**	13.43	68.97	83.25	90.35	94.18	101.35	96.31	93.33	-	98.61	99.92	93.61
	**45**	21.51	88.31	95.56	98.45	100.5	100.37	98.67	99.92	-	99.89	102.55	100.6
	**MPB**	17.55	69.90	76.77	80.91	84.06	85.45	79.63	77.49	71.91	100.26	101.60	99.35
**Parenterally vaccinated (Group 2)**	**47**	23.57	91.13	99.97	96.47	101.65	101.55	101.76	101.92	-	103.6	102.9	101.13
	**48**	19.26	85.96	99.08	101.14	101.63	100.1	101.36	101.33	-	102.18	103.35	100.6
	**49**	17.11	91.14	99.49	101.28	99.69	102.25	102.29	102.1	-	102.53	102.79	100.5
	**50**	21.6	93.57	100.69	101.23	100.67	101.13	101.48	100.25	-	102.28	103.07	100.52
	**51**	13.43	92.26	100.05	101.61	101.6	101.2	101.52	100.14	-	101.13	103.17	100.58
	**52**	14.32	91.49	98.07	100.52	101.75	101.46	101.47	102.19	-	99.25	103.83	99.96
	**MPB**	18.22	90.93	99.56	100.38	101.17	101.28	101.65	101.32	-	101.83	103.19	100.55
**Control (Group 3)**	**29**	13.61	9.68	11.14	6.66	19.51	13.74	-15.07	-9.24	71.8	-	-	-
	**36**	14.86	20.03	10.54	3.63	16.45	14.34	-15.96	-5.58	64.87	-	-	-
	**37**	11.54	11.78	3.84	-0.84	18.95	10.82	-7.42	-9.87	80.81	-	-	-
	**39**	20.16	15.01	21.11	22.46	23.37	18.64	7.2	2.55	74.57	-	-	-
	**46**	23.04	9.35	15.25	32.12	21.71	13.21	2.06	-4.36	56.79	-	-	-
	**MPB**	16.64	13.17	12.38	12.81	20.00	14.15	-5.84	-5.30	69.77	-	-	-

Table S3. *In silico* analysis of consensus between Ig-related mRNA and protein sequences of fox and dog. Alignments were performed using MUSCLE alignment on sequences of fox (VulVul2,0) and dog (CanFam3,1) genome annotations.

Species	Component	Gene ID	mRNA no.	Protein no.	Consensus [%]
Fox	FCAMR	112910995	XM_025987320.1	XP_025843105.1	
Dog	FCAMR	490267	XM_022420846.1	XP_022276554.1	87.65
Fox	FCAR	112909385	XM_025985292.1	XP_025841077.1	
Dog	FCAR	106559252	XM_014116440.1	XP_013971915.1	81.85
Fox	FCGR1A X1	112907678	XM_025982978.1	XP_025838763.1	
Dog	FCGR1A X1	403462	XM_022404314.1	XP_022260022.1	98.12
Fox	FCGR1A X2	112907678	XM_025982979.1	XP_025838764.1	
Dog	FCGR1A X2	403462	XM_014120558.2	XP_013976033.1	75.54
Fox	FCGR2B X1	112914296	XM_025991239.1	XP_025847024.1	
Dog	FCGR2B X1	100856270	XM_022415353.1	XP_022271061.1	92.86
Fox	FCGR2B X2	112914296	XM_025991241.1	XP_025847026.1	
Dog	FCGR2B X2	100856270	XM_022415354.1	XP_022271062.1	92.65
Fox	FCGR2B X3	112914296	XM_022415355.1	XP_025847027.1	
Dog	FCGR2B X3	100856270	XM_025991242.1	XP_022271063.1	85.76
Fox	FCGRT	112931350	XM_026014003.1	XP_025869788.1	
Dog	FCGRT	476414	XM_005616309.3	XP_005616366.1	99.44
Fox	FCGRT	112931350	XM_026014004.1	XP_025869789.1	
Dog	FCGRT	476414	XM_533618.7	XP_533618.2	99.15
Fox	FCMR	112910996	XM_025987321.1	XP_025843106.1	
Dog	FCMR X1	490266	XM_547385.6	XP_547385.3	91.35
Dog	FCMR X2	490266	XM_005622301.3	XP_005622358.1	89.54
Fox	JCHAIN	112923688	XM_026003517.1	XP_025859302.1	
Dog	JCHAIN	475166	XM_532398.6	XP_532398.2	98.39

Table S4. Individual numbers of IFN-γ specific SFU/10[6] cells counted at different time points pre- and post challenge after stimulation with 2.5 and 5.0 μg/mL antigen (RABV SAD L16), Numbers of counted SFU/10[6] cells were normalized to unstimulated controls.

		**Pre-Challenge**						**Post-Infection**												
	**dpv:**	**268**			**365**			**377**			**379**			**393**			**455**			
	**$c_{antigen}$ [μg/mL]:**	**2.5**	**5.0**	**3 ConA**	**2.5**	**5.0**	**3 ConA**	**2.5**	**5.0**	**3 ConA**	**2.5**	**5.0**	**3 ConA**	**2.5**	**5.0**	**3 ConA**	**2.5**	**5.0**	**3 ConA**	**non-stimulated controls**
	**Animal ID**																			
**Orally vaccinated (Group 1)**	**30**	18	18	373	0	5	350	-	-	-	15	50	630	60	38	1075	63	68	435	0
	**31**	38	18	648	25	68	778	-	-	-	60	88	540	53	38	1483	90	23	455	0
	**33**	8	28	388	120	220	1220	-	-	-	68	88	465	53	75	1788	153	125	305	0
	**34**	0	3	335	5	13	553	2508	2643	343	-	-	-	-	-	-	-	-	-	0
	**35**	15	10	490	45	40	830	-	-	-	110	90	688	50	33	1388	150	135	398	0
	**38**	18	10	378	20	15	460	-	-	-	100	83	710	128	408	2723	163	188	73	0
	**41**	0	0	628	33	8	435	1173	1068	320	-	-	-	-	-	-	-	-	-	0
	**42**	43	43	278	88	108	640	-	-	-	93	90	640	35	183	2155	130	108	735	0
	**43**	40	40	650	65	65	530	-	-	-	178	183	685	100	208	1985	158	143	673	0
	**44**	10	40	513	55	50	388	-	-	-	205	223	665	148	465	2215	100	98	573	0
	**45**	28	30	373	60	25	775	-	-	-	173	140	360	693	840	3265	125	133	715	0
	**mean**	20	22	459	47	56	633	-	-	-	111	115	598	147	254	2008	126	113	484	0
	**median**	18	18	388	45	40	553	-	-	-	100	90	640	60	183	1985	130	125	455	0
**parenterally vaccinated (Group 2)**	**47**	60	75	755	35	45	425	-	-	-	83	68	645	78	150	2750	20	63	278	0
	**48**	60	105	818	23	10	383	-	-	-	275	293	618	60	60	2180	93	145	825	0
	**49**	80	103	518	173	140	613	-	-	-	68	125	695	55	25	1803	83	108	448	0
	**50**	100	58	978	60	103	600	-	-	-	155	158	550	113	143	2018	183	165	630	0
	**51**	183	150	1045	88	53	418	-	-	-	50	73	715	83	63	2080	73	45	503	0
	**52**	28	18	230	73	80	275	-	-	-	170	188	568	55	63	1920	133	188	540	0
	**mean**	85	85	724	75	72	452	-	-	-	133	150	632	74	84	2125	97	119	537	0
	**median**	70	89	786	67	67	421	-	-	-	119	142	631	69	63	2049	88	127	521	0
**Control (Group 3)**	**29**	15	15	715	43	38	718	205	123	190	-	-	-	-	-	-	-	-	-	0
	**36**	15	8	735	20	38	483	1393	1213	358	-	-	-	-	-	-	-	-	-	0
	**37**	20	20	410	15	8	793	2548	2560	243	-	-	-	-	-	-	-	-	-	0
	**39**	10	8	353	25	15	555	665	528	405	-	-	-	-	-	-	-	-	-	0
	**46**	220	345	965	45	45	160	625	900	1575	-	-	-	-	-	-	-	-	-	0
	**mean**	56	79	636	30	29	542	1087	1065	554	-	-	-	-	-	-	-	-	-	0
	**median**	15	15	715	25	38	555	665	900	358	-	-	-	-	-	-	-	-	-	0

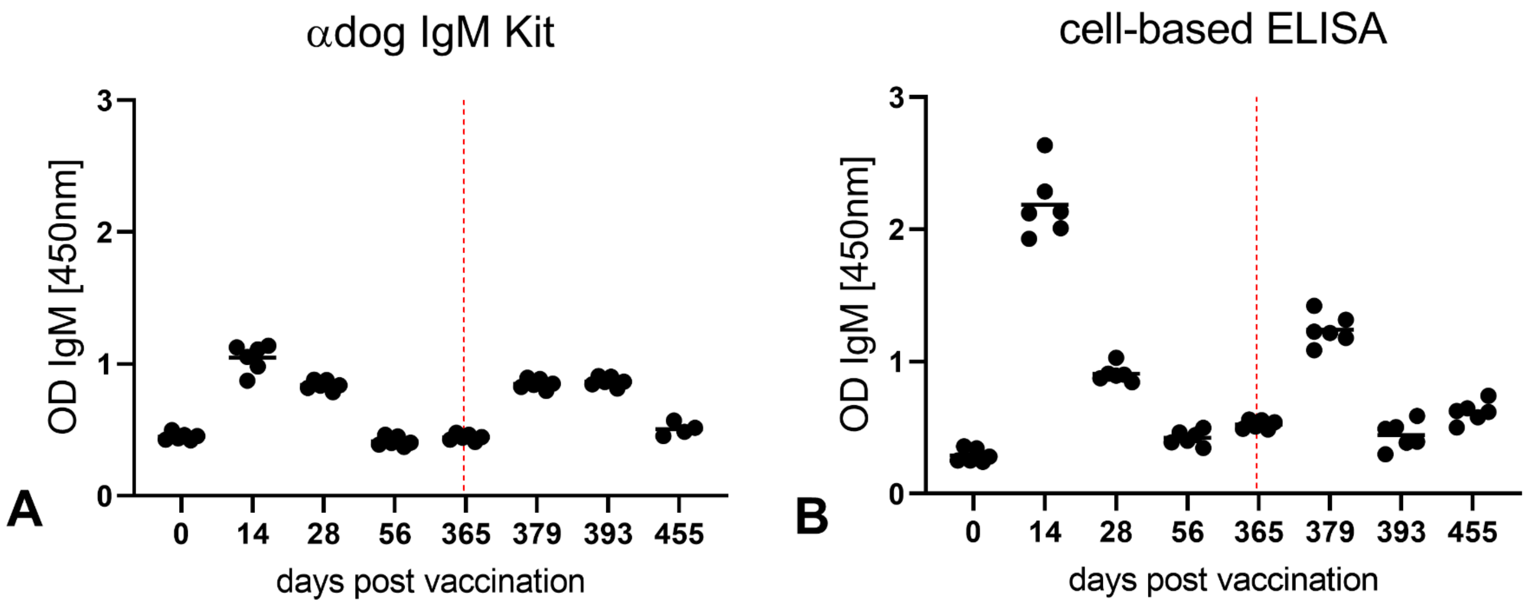

Figure S1. Comparison of RABV-specific IgM detection by a commercial dog-specific rabies virus IgM Antibody ELISA Kit and the cell-based ELISA. Cross-reactivity and specificity of IgM antibodies as measured by the cell -based ELISA established for foxes (**A**) was confirmed by a commercial dog-specific rabies virus IgM Antibody ELISA Kit (**B**) using 6 parenterally vaccinated animals selected at different time points dpv.
